# Survival and Recovery of the Pine-Tree Lappet *Dendrolimus pini* When Subjected to Simulated Starvation

**DOI:** 10.3390/insects11010067

**Published:** 2020-01-20

**Authors:** Adrian Łukowski, Dawid Adamczyk, Piotr Karolewski

**Affiliations:** 1 Institute of Dendrology, Polish Academy of Sciences, Parkowa 5, 62-035 Kórnik, Poland; dawidadamczyk.mog@gmail.com (D.A.); pkarolew@man.poznan.pl (P.K.); 2 Faculty of Forestry, Poznań University of Life Sciences, Wojska Polskiego 71c, 60-625 Poznań, Poland

**Keywords:** body mass, folivorous insect, instar, Lepidoptera, potentially invasive pest, Scots pine, starvation period

## Abstract

There are many reasons to study the survival and recovery of animals after starvation in simulated transport conditions or other passive dispersal methods. To do so, we chose *Dendrolimus pini*, an economically important pest of Scots pine with great potential in terms of passive dispersal outside its territory. In this work, we sought to answer the following questions: What is the maximum survival of different instar larvae after total starvation? Does access to dry tissues of the preferred host plant extend the lifespan of the larvae? Does the possibility of larvae recovery exist after starvation for various periods? We found that older larvae survived longer without food than younger larvae. Moreover, dry food did not extend the lifespan of the larvae. Our observations showed that insects were interested in food and tasted it at the beginning, but they did not feed on it for long. Furthermore, larvae recovery was indeed possible, and the time of starvation did not significantly affect this. We generally concluded that the *D. pini* larvae were characterized by the ability to survive without food for up to one month, which confirms that this species is able to survive long durations of transport to almost anywhere in the world.

## 1. Introduction

Insects are the most species-rich group of animals and it is not surprising that they are prominent invasive species, both in terms of numbers and their impacts [1,2,3,4]. Invasive species cause various ecological [5,6], social [2,7,8], and, in particular, economic problems [9,10,11]. Alien insect pest invasions can have significant negative ecological and economical effects on forests due to the slow growth of trees and their defoliation by pests. The prediction of forest insect invasions into new regions is an important issue [12,13], particularly for countries with high forest coverage [14]. Governments have taken steps to counteract the risk of the spread of alien pests to native plant species. Consequences of such invasions can be catastrophic for ecosystems and forest management (e.g., the introduction of the European gypsy moth (*Lymantria dispar* L.) in the United States in the 1860s [15]). Climate change and intensifying international trade are both factors contributing to the spread of insect species into new areas [10]. The risk of alien pest introduction is positively correlated with the trade of forest products [9] in areas with favorable climates and suitable host plants (i.e., environmental conditions that allow them to survive). Substantial economic savings and ecological safety could be achieved by collecting necessary data in carefully designed predictive studies. Quantification of the potential threat by such insects would benefit from ecological model-based risk analyses [16,17].

When modeling the potential distribution of an insect species, the current range of occurrence, climatic conditions, and the range of potential hosts are all often taken into account [18,19]. Little attention is devoted to other aspects of importance, such as the possibility of passive or active dispersion, introduction and acclimation, or human-assisted transfer. Non-native species are usually introduced into a new habitat by humans as a byproduct of the global trade and travel industries [3,20]. The prediction and monitoring of new invasions are technically feasible, but are not sufficiently funded [21]. Therefore, there is a major risk of insect introduction through trade, mainly due to the fact that regular border controls are only enacted for a narrow range of observed pathways [22]. Although important, whether species are able to survive long transport distances is rarely examined. Initially, the probability of successful invasion increases when a potentially invasive organism possesses complex mechanisms and behaviors which enable it to survive and recover after a long period of starvation. The most vulnerable forests are those that feature similar taxonomic compositions and climate conditions, such as the temperate forests of Europe, Asia, and North America [4], but it is readily apparent that these are far away from each other and not connected by ecological corridors. The plantation of some shared host plant species as ornamentals or along major routes of communication builds ecological corridors, thereby allowing alien species to enter and eventually invade new countries. There are few studies in this field, but each provides valuable information. For example, results achieved for Eurasian pests *Lymantria monacha* (L.) show that this species have the potential to be accidentally introduced via trade into other world areas due to fact that many North American plant species are suitable hosts [23] and that the larvae can survive longer without food than what was previously reported [24].

Insects garner food needed for their body maintenance, growth, and reproduction. They then experience spatiotemporal fluctuations in food supply, varying between years, seasons, and habitat quality [25,26,27]. When insects are incapable of feeding due to external food restrictions, they are considered to be starving. In contrast, fasting occurs when insects skip an occasion to consume for some intrinsic cause. Insects fast even when food is available, but mostly invest this time into other activities, such as those related to reproduction, migration, or molting [28]. Starvation eventually leads to starvation-induced mortality. Death by starvation as a result of intraspecific competition for food or varying abilities to resist starvation may be the main factors that control population dynamics [25,29]. Insects, however, do not die immediately when deprived of a host. Instead, they employ different strategies to survive and recover during periods of low or lacking food availability [26]. There are actually a variety of strategies, including nutrient storage, reducing energy use, or altering patterns of energy allocation [30], but dispersal from poor-quality sites is mainly for the purpose of regaining access to food [31]. Body size is assumed to play an important role in explaining differences in starvation resistance, which is often measured as time until death [32].

To study survival rates during starvation and recovery levels after a period of starvation as a result of simulated transport, we chose the model organism as it is a pest of great economic importance in areas of its natural occurrence and exhibits great potential in terms of passive dispersal opportunities outside of its territory. The pine-tree lappet *Dendrolimus pini* L. (family: Lasiocampidae) is native to Europe, with the exception of the United Kingdom, where it has quarantine status. In Poland, outbreaks usually take place in the central and western parts of the country [33,34,35]. The most recent outbreak in 2012–2014 covered an area reaching 131,000 ha in 2013 [36]. The natural range of *D. pini* mostly follows that of its primary host, the Scots pine, in Europe [13]. The larvae of pine-tree lappet moths feed gregariously on needles of mature trees, usually >20 years old [37]. Further, it is an economically important defoliator of pine forests in Europe [38,39] as European outbreaks are common and can last for seven to eight years. Repeated annual defoliation can result in tree mortality [40], and pine-tree lappet larvae may impact human health [41]. Dendrolimiasis (caterpillar-associated illness) is caused by contact with the hairs or haemolymph of living or dead larvae or cocoons and is characterized by maculopapular dermatitis, migratory inflammatory polyarthritis, migratory inflammatory polychondritis, and chronic osteoarthritis [41]. Our preliminary studies showed that this moth has many features of a potentially important invasive and bothersome species. For example, females lay an average of 298 eggs (±64 standard deviation) and larvae have a very low natural mortality rate, even under long-term lack of food conditions. It should also be considered that all developmental stages, including eggs, larvae, pupae, and adult insects, could easily use anthropogenic vectors. For example, as we observed personally, although eggs were normally laid on needles, they could also be deposited onto other materials, like wood, containers, pallets, ships, etc. Thus, there is the potential for the species to move via the transport of forest products within the EU, Europe, and globally. For example, Europe is the main trading partner with North American and Asian countries, meaning that the European species have a disproportionately greater opportunity to be transported and become established in these areas [12].

The aim of this study was to answer the following questions: (1) What is the maximum survival of different instar larvae after total starvation? (2) Does access to dry tissues of the preferred host plant extend the lifespan of the larvae? (3) Does the possibility of larvae recovery exist after starvation for various periods? We hypothesized that (1) the larvae of later instars survive longer than younger ones, (2) access to the preferred host plant dry tissues, which is possible during accidental transport (i.e., dry needles among logs of wood), significantly extends the life of the larvae, and (3) recovery is possible, but with a higher mortality rate. In this work, we determined several important aspects related to the survival of the pine-tree lappet, the serious pest of *Pinus* stands, with the results being of great practical importance. Precise predictions of the potential geographical distribution of the pine-tree lappet requires thorough knowledge in the context of the possibility of passive dispersal and survival rates during starvation (e.g., during long periods of larvae transport with wood or other goods) in order to evaluate the risk of invasion by this species on a global scale.

## 2. Materials and Methods

### 2.1. Insects and Plant Material

In this study, we used L1 and L3 instar larvae. The individuals of the last instar larvae, which were used to start the rearing as parents of laboratory stock, were derived from nature, specifically from stands in the Gołąbki Forestry District, State Forests, in Poland (52.6326, 17.7731). All experiments were carried out with the full protection of human health due to the presence of strong allergens in larvae hairs [41]. Additionally, because of the high risk of damage to neighboring stands, all larvae and adult insects were eliminated at the end of the experiment by transferring them to a deep freezer for one week. This study was carried out in strict accordance with the ethical standards regarding entomological research. The collection and breeding of insects in Poland were allowed without additional permission.

Twenty similarly sized young trees of Scots pine (*Pinus sylvestris* L.) were employed to feed the larvae according to the established schedule (see subsequent section). On the day of food exchange, we supplied fresh shoots with one-year-old needles obtained from a randomly selected tree, which was different each time.

### 2.2. Experimental Design and Measurements

In July 2019, we chose 20 freshly emergent adults (10 pairs from many possible to choose; F0 generation; larvae from nature), which, after the necessary biometric measurements (e.g., body mass) were completed, were randomly combined into pairs (mating usually occurs approximately one hour after placing individuals in a container). All pairs were allocated to a plastic container with a fresh shoot of Scots pine in medium to oviposition. As the moths do not feed during the adult stage, there was no need for artificial feeding. Pine-tree lappet females laid eggs only at night for the following few days, so on the second day of laying eggs, we collected all laid eggs and determined the masses. Eggs from each female were placed together onto a Petri dish in order to observe the process of hatching. The larvae were then randomly separated into three groups according to the three different experiments.

#### 2.2.1. Experiment I: What Is the Maximum Survival of Different Instar Larvae after Total Starvation?

The newly hatched larvae, which had already eaten the chorion, were weighed and transferred to a larger box. From the whole neonate larval group, we randomly selected 60 individuals (30 control and 30 treatment), which were moved individually to 55-mm Petri dishes with vents after weighing. The initial body masses were measured using the Radwag (AS 82/220.R2) analytical balance (up to 0.01 mg). The larvae were kept under the following conditions: 23 °C, relative humidity of 60%, and light-emitting diode light similar to natural (day/night, 16:8 h). “Control” larvae were fed *ad libitum* until the death of the last starved larva. *Ad libitum* feeding involved supplying a portion of fresh needles that slightly exceeded the daily larval requirement for food. Food was replaced with a new food supply as needed, usually once a day. Larval mortality was recorded every day at 09:00. This was performed by observing, without touching, the dishes so as to not expose the larvae to additional stress. When observation indicated that the larva was already dead or almost dead, the dish was opened gently to touch it with tweezers to ascertain death. The experiment ended when the last larva died.

To avoid the impact of intraspecific competition (i.e., an uneven number of larvae reared in one box), we split the remaining group of neonate larvae evenly (30 larvae per box). In these boxes, the larvae were fed *ad libitum* until most larvae reached the L3 instar. Then, the larvae that completed the molting process the previous night (identified by the lighter body/hair color) were selected from the containers and placed into a larger container (containing larvae from various boxes). We drew 60 larvae from this container (30 control and 30 treatment), which were moved individually to 55-mm Petri dishes with vents after weighing. “Control” larvae were fed *ad libitum* until the last starved larva died. The larvae were kept under the same conditions as described before. Larval mortality was recorded every day at 09:00 in the same way as presented earlier.

#### 2.2.2. Experiment II: Does Dried Food Extend the Lifespan of the Larvae?

We drew another 90 larvae that reached the L3 instar from common containers (see above preparation instructions), which were moved individually to 55-mm Petri dishes with vents after weighing. “Control” larvae (*n* = 30) were fed *ad libitum* until the last starved larva died. “Dried food”-variant larvae (*n* = 30) received approximately 0.05 g of air-dried one-year-old pine needles (needles were air-dried in an envelope for seven days before the experiment; the average water content of the needles was 5.06% ± 1.21% SD) and 0.05 g of bark of Scots pine at the very beginning of the experiment. The remaining larvae (*n* = 30) did not receive any food. The larvae were kept under the same conditions as described in Experiment I. Larval mortality was recorded every day at 09:00 according to the same method described earlier.

#### 2.2.3. Experiment III: Is There a Possibility of Recovery after Starving Larvae for Various Periods of Time?

We approximately drew another 450 larvae that reached the L3 instar from common containers (see above preparation instructions). The first 20 were marked as controls, which were moved individually to 55-mm Petri dishes with vents after weighing and fed *ad libitum* for the next two weeks. The remaining larvae were kept in plastic boxes with volumes of 1 L (roughly 30 per box) and starved. Larval mortality in the plastic boxes was recorded every day at 10:00 and dead larvae were removed immediately. The results of these observations are not presented in the results section in order to avoid showing similar results to those obtained in Experiment I. From this group, after 3, 7, 10, and 14 days of starvation in the plastic boxes, 20 larvae from each period were randomly selected, transferred individually to 55-mm Petri dishes with vents, and fed *ad libitum* for the next two weeks. All larvae in this experiment were kept under the same conditions throughout as described in Experiment I. Larval mortality was recorded every day at 09:00 according to the same methods presented above.

### 2.3. Statistical Analyses

Survival analysis (the log-rank test) was applied to determine the differences in survival of the larvae between treatments over time in each experiment. A one-way analysis of variance (ANOVA) model was employed to compare the initial mass of the larvae in each experiment. Prior to all analyses, normal distributions were verified using the Shapiro–Wilk test. The data regarding the masses and lethal times were expressed as means with standard errors of the mean (±SE). All calculations were performed using JMP software (SAS Institute Inc., Cary, NC, USA).

## 3. Results

**Figure 1:**
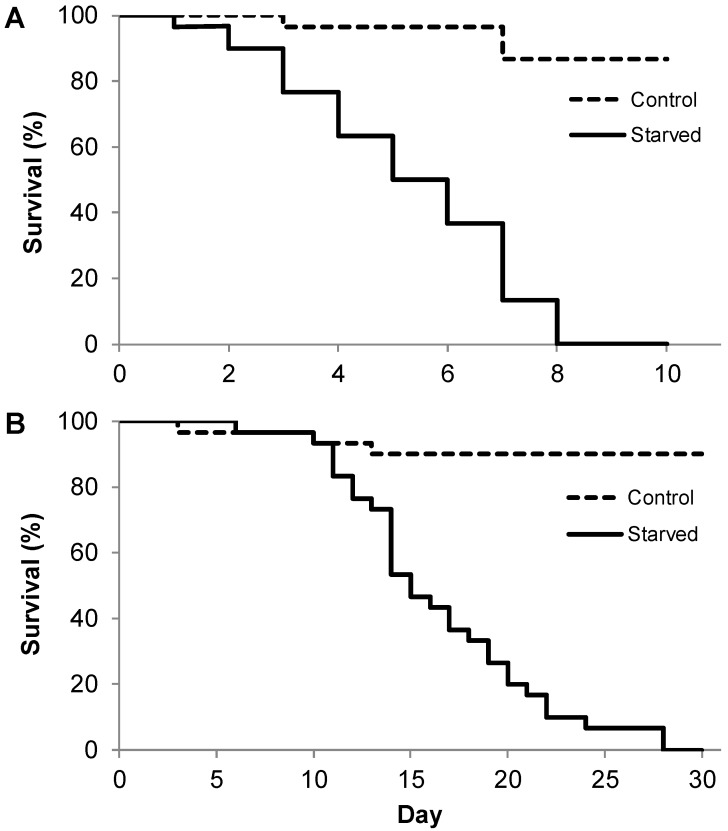
The survival analysis (log-rank test) of L1 ((**A**); *p* < 0.0001) and L3 ((**B**); *p* < 0.0001) pine-tree lappet *Dendrolimus pini* larvae for two variants of breeding, i.e., those fed *ad libitum* (control) and those without food (starved).

We found no significant differences in initial larval mass of the control variant compared to the larvae subjected to simulated starvation for both L1 (F_1.58_ = 0.0280; *p* = 0.8677), with the control mass being 0.00313 (±0.00011) g and the starved mass being 0.00312 (±0.00012) g, and L3 larvae (F_1.59_ = 0.2985; *p* = 0.5869), with the control mass being 0.259 (±0.012) g and the starved mass being 0.252 (±0.009) g. In Experiment I, the maximum survival time during total starvation of the L1 and L3 instar larvae was tested, showing that the larval instar significantly affected mortality when undergoing both starvation and natural conditions (Figure 1). The maximum lifespan of the starved L1 larvae was 8 days (Figure 1A), with L3 larvae surviving up to 28 days (Figure 1B). The larvae reached a mortality rate of 50% after 6 and 16 days for L1 and L3, respectively. After a period of experimentation (i.e., eight days), the control L1 larvae, i.e., those fed *ad libitum*, had a mortality rate of 13.3%. In contrast, the control L3 larvae had a mortality of 3.3% after a period of the same as L1 larvae (i.e., eight days) and 10% after a period of 28 days. The log-rank survival test clearly demonstrated that there were significant differences in survival between the control and starved variants for both instar larvae.

**Figure 2:**
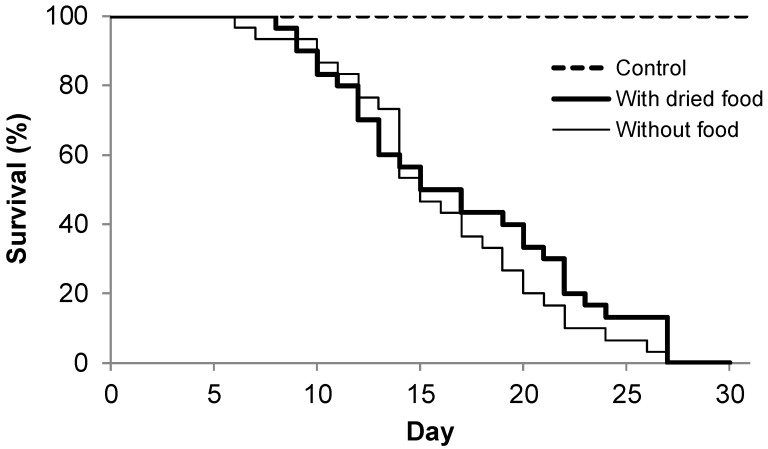
The survival analysis (log-rank test; *p* < 0.3922) of L3 pine-tree lappet *Dendrolimus pini* larvae for three variants of breeding, i.e., with food (control), with a mix of dried food (with dried food; see Materials and Methods), and without food. The log-rank test was carried out only for the “with dried food” and “without food” treatments.

We found no significant differences in the initial larvae mass of the control variant compared to the larvae subjected to both simulated starvation treatments (F_2.87_ = 0.3681; *p* = 0.6931), where the control larvae had a mass of 0.257 (±0.012) g, the larvae fed dried food had a mass of 0.258 (±0.013) g, and the starved larvae had a mass of 0.254 (±0.012) g. In Experiment II, we checked whether access to dry food contributed to life extension of larvae using L3 larvae as a model (Figure 2). The log-rank survival test showed that there were no significant differences in survival between the larvae fed dried food and those with no food (starved). The maximum lifespan for both variants was 28 days. Similarly, half-level mortality of the larvae was observed at 16 days for both variants. After a period of experimentation (i.e., 28 days), the control larvae, i.e., those fed *ad libitum*, all survived (Figure 2).

**Figure 3:**
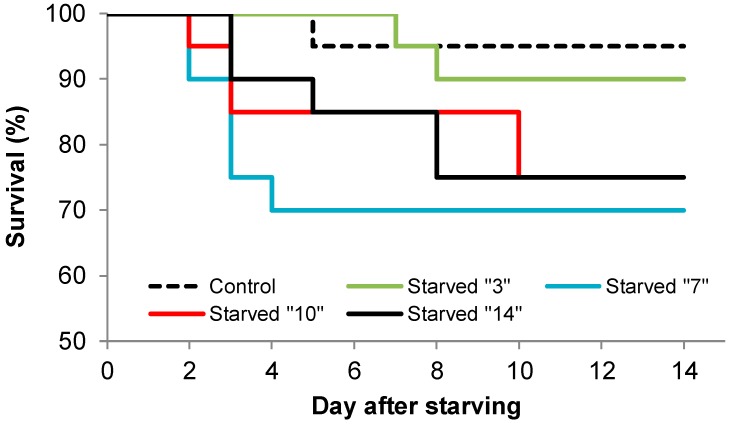
The survival (log-rank test; *p* < 0.1716) of L3 pine-tree lappet *Dendrolimus pini* larvae fed *ad libitum* (control) and for four variants of breeding, i.e., starved for 3, 7, 10, and 14 days and then fed *ad libitum* for the next 14 days. Survival during the periods in which the larvae were subjected to starvation is not shown in the figure. Only surviving larvae were taken to observe the recovery phenomenon.

In this experiment, we also observed no significant differences in the initial larval masses between all treatments (F_4.95_ = 1.0887; *p* = 0.3666), where the control larvae had a mass of 0.253 (±0.019) g, the larvae starved for 3 days had a mass of 0.273 (±0.017) g, those starved for 7 days had a mass of 0.289 (±0.014) g, those starved for 10 days had a mass of 0.252 (±0.019) g, and those starved for 14 days had a mass of 0.255 (±0.015) g. Only surviving larvae were taken to observe the recovery phenomenon (see Section 2.2.3). Survival during the periods in which the larvae were subjected to starvation is not shown in Figure 3 but can be roughly read from Figure 1. In Experiment III, where we determined whether larvae that were starved for various durations could recover, we established that, in each of the treatments, some of the larvae survived the next 14 days until they again received *ad libitum* feeding (Figure 3). Almost all (95%) of the control larvae fed *ad libitum* survived throughout the period of the experiment (i.e., 14 days). The log-rank survival test showed that there were no significant differences in survival between the control larvae and the rest of the treatments. The highest levels of survivability (except controls) were characterized by the larvae subjected to simulated starvation for just three days. Calculations showed that larvae that were starved for 3 days, then fed and nevertheless died, had an average mortality time of 7.5 ± 0.5 days, larvae that were starved for 7 days had an average mortality time of 2.8 ± 0.3 days, larvae with the 10-day treatment had an average mortality time of 5.6 ± 1.8 days, and those with treatment for 14 days had an average mortality time of 5.4 ± 1.1 days.

## 4. Discussion

### 4.1. Lifespan of Larvae During Starvation

In this research, we determined that older larvae survived longer without food (up to 28 days) than younger larvae, therefore confirming the first hypothesis. Females lay ca. 300 eggs on branches, trunks, bark, and needles in aggregations of up to 100 eggs [34]. Thus, newly emerged larvae of pine-tree lappet moths must be established quickly or die. In this species, the first instar larvae can be wind-dispersed over significant distances and can reach uninfested trees, a process known as ballooning. Furthermore, a large body size is more efficient in terms of energy use (*relative efficiency hypothesis*), but requires more energy to be sustained, which presents difficulty when starving (*absolute energy demand hypothesis*; [42]). It is broadly postulated that a greater body mass affords greater resistance to starvation [32,43]. More advanced insects are suggested to survive longer under starvation conditions than smaller ones (despite the greater absolute energy requirements of large individuals), given the same percentages of body mass as energy reserves [43]. Relative metabolic rate scales are inversely related to body mass. Thus, less energy is expended for each additional unit of body mass. Our results support the relative efficiency hypothesis, namely that a greater body mass of L3 instar larvae compensates for the greater absolute energy demand, thus allowing them to survive longer in comparison with L1 instar larvae.

Regarding the second hypothesis, which was rejected, it was expected that access to dry tissues of the preferred host plant would significantly extend the life of the larvae. It turned out that the larvae fed dried food and those without any food (starved) exhibited similar maximum lifespans. Our observations showed that insects were interested in this food and tasted it at the beginning, but ultimately did not feed on it for long, which was identified by a small amount of frass. Larvae did not prefer this type of food due to a significant decrease in the quality of the food. Leaf and needle chemistry along with structural defense, such as toughness, determine food quality and the resulting food preferences of folivores [44,45]. Among many different components affecting the chemical quality of food, water content is one of the most important traits affecting feeding preference, survival, and larval performance. Phytophagous insects normally feed on foods with optimal water contents, which is recognized as adequate food hydration, therefore enabling unwavering support for physiological processes in insect organisms [46]. “Optimal” for a particular insect species refers to how species differ in requirements for water contents in their food [47]. Any deviation, decrease, or even increase in water content significantly affects the insect, e.g., the growth and survival of the pine sawfly *Neodiprion sertifer* (Geoffroy), and the species is therefore adversely influenced by a higher water content [48]. Under drought stress, for example, plants exhibit reductions in water content and increases in leaf nitrogen and soluble sugars, but also exhibit defense compounds that are associated with changes that occur during wilting, alongside less dilution of other components [49]. Dry needle or leaf tissues are speculated to be completely unsuitable food for non-adapted specimens, and attempts to feed on such food leads to negative consequences, such as clogging of the digestive system.

### 4.2. Ability to Recover

The assessment of *D. pini* larval survival after starvation and a period of recovery provided new insights into the effects of starvation on this species and is one step toward learning more about the biology of this species [35]. This study is the first to examine the effects of starvation and recovery periods on the survival rates of this species. Older larvae were more resistant to starvation because they survived relatively long periods. Contrary to the expectations expressed in the third hypothesis, the recovery of larvae was possible, and survival was significant. Starved L3 larvae tended to recover well when food became accessible, with high recovery levels. Although nonsignificant, a visible trend was observed showing that the longer the starvation period, the lower the survival rate. However, even after two weeks, this rate was considered to be high (70%). In addition, it is logical to assume that insufficient food would cause the resulting adults to be smaller than normal and/or would prolong the duration of larval development. Weiss et al. [50] concluded that as food became limited, the duration of larval development increased, adult numbers in nature diminished, the sex ratio deflected, and the survival of adults reduced. It was concluded that the starvation period would affect other life history traits of *D. pini*, e.g., reproduction or development. In other words, it was speculated that the larvae would not be able to make up for the losses resulting from the lack of increase in body mass after such a period of starvation. For example, the same trend may be observed in the beetles *Tribolium castaneum* (Herbst), *Rhyzopertha dominica* (F.), and *Sitophilus oryzae* (L.), specifically, that the longer the starvation period, the less females lay eggs after recovery [51]. It was not excluded that for this moth, body mass is also correlated with fecundity, as confirmed for many other moth species [52]. To sum up, these results allowed us to establish that the investigated species has an additional feature characteristic of potentially invasive insect pests, i.e., it recovers well when transferred to food after starvation.

### 4.3. Application Aspects and Future Directions

As shown, pine-tree lappet moth larvae are characterized by the ability to survive without food for up to a month, therefore confirming that this species could survive long passive dispersal periods to almost anywhere in the world via anthropogenic vectors. *Dendrolimus pini* has great potential to be transported via the trade industry. There are many requirements that must be met by a species in order to successfully invade new land. In the case of alien insects, survival of transport, new host plant acceptability, and suitable climatic conditions are highly significant elements of the further dispersion of a species within a new environment [16,23,53]. The most important factor of a biological invasion is the spreading dynamic. Usually, the spread of insects is characterized by stratified dispersal (e.g., the establishment of new colonies far from the moving population front or growth of individual colonies), which includes natural short-distance spread by flight [54]. Long-distance insect spread can be enhanced by the anthropogenic transport of infested material, like wood or plants (e.g., firewood, biomass, nursery material) [22,55]. The majority of alien insect species were accidentally transported with imported goods or by travelers [12], for example, the lesser budmoth, *Recurvaria nanella* (Denis and Schiffermüller) with living plants [12,56] and the emerald ash borer, *Agrilus planipennis* Fairmaire, which arrived in infested solid wood-packing material [55,57]. In the aforementioned examples, insects experienced easier dispersion because they traveled with their native food. “Hitchhiking” is another available path of unintentional movement of species, usually connected with a journey without food (period of starvation) by car, sea containers, trains, ships, etc. [12].

In this research, we found that the larvae survived a relatively long time, leading to the conclusion that this species could successfully disperse even via “hitchhiking”. In addition, it should be considered that other developmental stages, such as eggs, pupae, or adult insects, could also easily use anthropogenic vectors. For example, as observed, although eggs are normally laid on needles, they could also be deposited onto other material, like wood, containers, pallets, ships, etc. Moreover, the egg incubation period lasts an average of 14 to 25 days, pupae typically require 18 to 35 days, and adult insects live for roughly 10 days [58]. On the other hand, the effect of starvation during transportation on infection interactions is very important. This should be taken into account, particularly for vertically transmitted covert infections (e.g., baculoviruses), because they also infect the *Dendrolimus* genus [59]. Studies by Kasianov et al. [60] showed that even four days of starvation led to the activation of covert viral infection in *Lymantria disapr* larvae. Viral disease could significantly regulate the density of population. Thus, if a population of *D. pini* contained covert pathogens, starvation would lead to a significant mortality rate after activation of the infection to the acute stage.

Ecologists usually utilize niche modeling to predict locations of high compliance with insects’ environmental requirements and preferred food [61], but these models do not take into account the dispersion capabilities of the species. All of these considerations led us to the general conclusion that when we recognize a species as pursuing a potential invasion of a new area, we should first make a comprehensive assessment of its survival in different disperse conditions (especially under the stress of starvation) aside from the obvious studies of acceptance of potential host plants and the environmental requirements of the insect. As a summary, we would like to highlight a few other related issues. It should be clearly stated that the larval survival is relatively relevant, but the probable fertility of the corresponding adults is more essential here. Therefore, as a next step, we plan to address this issue. Moreover, the unwanted introduction of large caterpillars is an uncommon event, even in times of increased chance of alien species introduction. Finally, at the start of the winter season, larvae move down from the trees and remain dormant in forest litter or soil during the entire winter season (from November to March [35]). The diapausing larvae in this species could survive even longer than the L3 larvae in the present experiment.

Generally, scaling the metabolic and reserve exhaustion ratios sufficiently explains the lengthier fasting periods in larger organisms related to smaller ones [62]. Moreover, positive connections between body size and starvation resistance were observed within different species groups, for example, in moths [43], beetles [63], mosquitoes [64], and backswimmers [32]. However, the opposite trend was also noted, e.g., in bumble bees [65]. In the future, the above-mentioned relationships and their importance in supporting the biological invasion of insects should be checked by meta-analysis. Ideally, body size parameters of insects that successfully invaded new areas should be compared with survival times (also during starvation) to determine the success of the invasion. Following this, it would be necessary to cover the outbreak occurrence of a larger number of pests with high economic importance, similarly to this study of *D. pini*. We plan to extend our research in many aspects concerning the starvation process and its influence on the physiology, resistance, and response of adult insects (regarding fertility, oviposition, survival, and performance of their offspring).

### 4.4. Study Limitations

First, this experiment was a typical laboratory experiment, where other additional factors that may prove to be important with respect to the maximum survivability were omitted (according to the *ceteris paribus* principle). For example, temperature and humidity were not taken into account. Generally, the survival times of newly emerged larvae are longer at lower air temperatures and higher humidity levels [24,66,67], but there are many exceptions to this [68]. Although it is possible that the observed tolerance to starvation depended on the experimental conditions used, the limited data on *D. pini* larval survival suggest that this was not the case.

Second, we used relatively small containers in this experiment, which limited the possibility of larvae starting intensive food searches on the one hand [26], but on the other, minimized larval energy loss [28,32]. A common response to starvation is initially to increase activity in order to find food, but if the host plant is not identified, there is the impetus to reduce activity to conserve energy. Therefore, although this probably influenced the observed survival result, it was probably insignificant due to the ability to move, even though the container was small.

Third, this study did not consider whether the population of *D. pini* contained covert pathogens. The effect of starvation on infection interactions is important, mainly due to baculoviruses that can infect *Dendrolimus* [59].

Finally, larval stress was diminished during this experiment to the necessary minimum, e.g., no natural enemies were present. Therefore, it is difficult to find a simple conversion of the results obtained from this experiment for research that would be carried out under field conditions, e.g., during a simulation of passive dispersal with wood or other goods transport. Starvation, however, has negative effects and is very stressful in and of itself. Thus, it can be assumed that an internal mechanism was at work here, where the initial stress enhanced performance and further (chronic) stress impaired it [26,69].

## 5. Conclusions

As a summary, we answered the questions from the Introduction section as follows:

*(1) What is the maximum survival of different instar larvae after total starvation?* We found that older larvae (L3) survived longer without food than newly hatched larvae (L1). The maximum lifespan of the starved L1 larvae was more than a week and L3 larvae survived almost a month. The half-level mortality of the larvae was reached at approximately one and two weeks for the L1 and L3 larvae, respectively.

*(2) Does access to dry tissues of the preferred host plant extend the lifespan of the larvae?* There were no significant differences in survival between the larvae fed dried food and the larvae fed no food (starved). Generally, access to dry food did not contribute to prolongation of the life of L3 larvae. Our observations showed that insects were interested in the food and tasted it at the beginning, but ultimately did not feed on it for long.

*(3) Does the possibility of larvae recovery exist after starvation for various periods?* Recovery after various periods of starvation was possible and the effect on survival was significant. We generally conclude that *D. pini* larvae are characterized by the ability to survive without food for up to a month, thereby confirming that this species could survive long migration periods to almost anywhere in the world using anthropogenic vectors.
